# c-Rel is dispensable for the differentiation and functional maturation of M cells in the follicle-associated epithelium

**DOI:** 10.1016/j.imbio.2016.09.008

**Published:** 2017-02

**Authors:** Anuj Sehgal, Atsushi Kobayashi, David S. Donaldson, Neil A. Mabbott

**Affiliations:** a The Roslin Institute and Royal (Dick) School of Veterinary Sciences, University of Edinburgh, Easter Bush, Midlothian, EH25 9RG, UK; b Laboratory of Comparative Pathology, Graduate School of Veterinary Medicine, Hokkaido University, Sapporo, Japan

**Keywords:** Anxa5, annexin A5, FAE, follicle-associated epithelium, FDC, follicular dendritic cell, GALT, gut-associated lymphoid tissues, IHC, immunohistochemistry, Marcksl1, myristoylated alanine-rich C-kinase substrate-like protein 1, NF-κB, nuclear factor-κB, RANK, receptor activator of nuclear factor-κB, RANKL, receptor activator of nuclear factor-κB ligand, Scg5, secretogranin V, SED, sub-epithelial dome, LT, lymphotoxin, UBD, ubiquitin D, UEA-1, *Ulex europaeus* agglutinin-1, c-Rel, M cells, Follicle-associated epithelium, Peyer’s patches, RANKL

## Abstract

M cells reside within the follicle-associated epithelium (FAE) overlying the gut-associated lymphoid tissues. These unique phagocytic epithelial cells enable the mucosal immune system to sample antigens within the lumen of the intestine. The differentiation of M cells from uncommitted precursors in the FAE is dependent on the production of receptor activator of nuclear factor-κB ligand (RANKL) by subepithelial stromal cells. The ligation of a variety of cell surface receptors activates the nuclear factor-κB (NF-κB) family of transcription factors which in-turn induce the transcription of multiple target genes. RANKL-stimulation can stimulate the nuclear translocation of the NF-κB subunit c-Rel. We therefore used c-Rel-deficient mice to determine whether the differentiation and functional maturation of M cells in the Peyer’s patches was dependent on c-Rel. Our data show that c-Rel-deficiency does not influence the expression of RANKL or RANK in Peyer’s patches, or the induction of M-cell differentiation in the FAE. RANKL-stimulation in the differentiating M cells induces the expression of SpiB which is essential for their subsequent maturation. However, SpiB expression in the FAE was also unaffected in the absence of c-Rel. As a consequence, the functional maturation of M cells was not impaired in the Peyer’s patches of c-Rel-deficient mice. Although our data showed that the specific expression of CCL20 and ubiquitin D in the FAE was not impeded in the absence of c-Rel, the expression of ubiquitin D was dramatically reduced in the B cell-follicles of c-Rel-deficient mice. Coincident with this, we also observed that the status of follicular dendritic cells in the B cell-follicles was dramatically reduced in Peyer’s patches from c-Rel-deficient mice. Taken together, our data show that c-Rel is dispensable for the RANKL-mediated differentiation and functional maturation of M cells.

## 1 Introduction

The mucosal immune system in the intestine provides the first line of defence against orally-acquired pathogens. However, in order to initiate an effective mucosal immune response, the antigens within the lumen of the intestine must first be transported across the intestinal epithelium into the gut-associated lymphoid tissues (GALT) comprising primarily of the Peyer’s patches, appendix, colonic and caecal patches and isolated lymphoid follicles. The specialized follicle-associated epithelium (FAE) which overlies the GALT contains a unique population of epithelial cells, termed M cells (for review see (Mabbott et al., 2013)). These highly phagocytic cells are specialized for the transfer of a broad range of particulate antigens and microorganisms across the FAE into the GALT by a process termed *transcytosis*. Following transport by M cells the antigens are rapidly released into their underlying basolateral pockets where they are processed by mononuclear phagocytes (Wang et al., 2011, Lelouard et al., 2012). The transcytosis of antigens across the FAE by M cells is an important initial step in the induction of efficient mucosal immune responses against certain pathogenic bacteria (Hase et al., 2009, Kanaya et al., 2012) as well as the commensal bacterial flora (Rios et al., 2015).

There has been much effort in recent years to identify the factors which regulate M-cell differentiation and function. Since certain bacterial and viral pathogens, as well as prions, appear to exploit M cells to infect host tissues (Heppner et al., 2001, Fujimura et al., 2004, Westphal et al., 2008, Donaldson et al., 2012, Nakato et al., 2012, Tahoun et al., 2012, Gonzalez-Hernandez et al., 2014), a thorough understanding of these factors may aid the development of novel strategies to prevent infection. Stromal cells with the sub-epithelial dome (SED) region of the GALT play a critical role in inducing M-cell differentiation in the FAE by producing the cytokine named receptor activator of NF-κB ligand (RANKL) (Taylor et al., 2007). Enterocytes in the FAE express the RANKL receptor (receptor activator of NF-κB; RANK) and are stimulated by RANKL to differentiate into M cells. Furthermore, *in vivo* neutralization of RANKL blocks M-cell differentiation, and Peyer’s patches from RANKL-deficient mice lack M cells (Knoop et al., 2009). The temporary depletion of M cells after RANKL-neutralization also significantly reduces susceptibility to oral infection with prions (Donaldson et al., 2012), norovirus or reovirus (Gonzalez-Hernandez et al., 2014), and prevents uptake and toxicity after oral exposure to botulinum toxin A (Matsumura et al., 2015).

The fate and terminal differentiation of distinct intestinal epithelial cell lineages from their uncommitted precursors is dependent on their intrinsic expression of one or more specific transcription factors during their development. For example, Sox9 expression is required for Paneth cell maturation (Bastide et al., 2007, Mori-Akiyama et al., 2007), neurogenin 3 is required for enteroendocrine cell maturation (Jenny et al., 2002) and Klf4 is required for the terminal differentiation of goblet cells (Katz et al., 2002). In a previous study we identified a co-expressed transcriptional signature which contained genes which were specifically expressed in the FAE and by M cells (Kobayashi et al., 2013). Analysis of the transcription factor binding site motifs in the promoter regions within this cluster of genes indicated that they shared a transcriptional programme, and suggested that motifs for the nuclear factor-κB (NF-κB) family of transcription factors were significantly enriched (Kobayashi et al., 2013). The NF-κB family of transcription factors consists of five members: NF-κB1 (p50), NF-κB2 (p52), RelA (p65), RelB and c-Rel. These subunits form homodimeric or heterodimeric complexes, and each shares a highly conserved region designated as the Rel domain, which is responsible for DNA binding and dimerization. A variety of cell stimuli activate NF-κB transcription factors which in-turn induces the transcription of multiple target genes (May and Ghosh, 1998). For example, RANKL-stimulation can induce the nuclear translocation of c-Rel (Ruocco et al., 2005), and *in vitro* studies show that RANKL-RANK stimulation in RAW cells triggers a cascade of intracellular events which induces the DNA binding of NF-κB complexes consisting of NF-κB1, RelA and c-Rel (Kang et al., 2003). The NF-κB subunits RelA and RelB play a critical role in the development of secondary lymphoid tissues, including Peyer’s patches. Furthermore, the development of Peyer’s patches in RelA and RelB is blocked (Yilmaz et al., 2003, Alcamo et al., 2002). As a consequence of this deficiency it is not possible to study the role of RelA and RelB in the FAE and the M cells within it using RelA- or RelB-deficient mice since they lack Peyer’s patches. However, the formation of secondary lymphoid tissues including Peyer’s patches in c-Rel^−/−^ mice, in contrast, is not adversely affected (Liou et al., 1999) and were used here to determine whether c-Rel expression was essential for the differentiation and functional maturation of M cells in Peyer’s patches.

## 2 Materials and methods

### 2.1 Mice

Six- to 8-week old c-Rel-deficient (c-Rel^−/−^) mice (Liou et al., 1999) or C57BL/6J mice were used as wild type (WT) controls throughout this study. All studies using experimental mice and regulatory licences were approved by both The Roslin Institute’s and University of Edinburgh’s ethics committees. All animal experiments were carried out under the authority of a UK Home Office Project Licence within the terms and conditions of the strict regulations of the UK Home Office ‘Animals (scientific procedures) Act 1986’. Where necessary, anaesthesia appropriate for the procedure was administered, and all efforts were made to minimize harm and suffering. Mice were humanely culled using by a UK Home Office Schedule One method.

### 2.2 *In vivo* assessment of M cell-mediated transcytosis

Mice were given a single oral gavage of 1 × 10^12^ of Fluoresbrite Yellow Green labelled 200 nm microbeads (Polysciences, Eppelheim, Germany) in 200 μl PBS. Mice were culled 24 h later and Peyer’s patches removed and snap-frozen at the temperature of liquid nitrogen. Serial frozen sections (10 μm in thickness) were cut on a cryostat and counterstained with DAPI. Images of follicles from three Peyer’s Patches per mouse (*n* = 4 mice/group) from at least 6 non-sequential sections (at least 100 μm apart) were acquired using Nikon Eclipse E400 fluorescent microscope using Micro Manager (http://www.micro-manager.org). The number of beads and the area of lymphoid tissue in each section were determined using ImageJ (http://imagej.nih.gov/ij) and the bead density calculated. Tissue auto-fluorescence was subtracted from displayed images using ImageJ.

### 2.3 Immunohistochemistry (IHC)

For whole-mount staining, Peyer’s patches were fixed with BD Cytofix/Cytoperm (BD Biosciences, Oxford, UK) and immunostained with rat anti-mouse GP2 mAb (MBL International, Woburn, MA). Tissues were subsequently stained with Alexa Fluor 488-conjugated anti-rat IgG Ab (Invitrogen, Paisley, UK), rhodamine-conjugated *Ulex europaeus* agglutinin I (UEA-1; Vector Laboratories Inc., Burlingame, CA) and Alexa Fluor 647-conjugated phalloidin (ThermoFisher Scientific, Paisley, UK).

For analysis of tissue sections, intestinal pieces containing a Peyer’s patch were snap-frozen in liquid nitrogen or fixed in 4% paraformaldehyde before embedding in optimal cryostat temperature medium (VWR, Leighton Buzzard, UK). Tissue sections (5 μm) were immunostained with the relevant primary antibody: rat anti-mouse CD21/35 (clone 7G6; BD, Biosciences); rat anti-mouse B220 mAb (clone RA3-RB2, Caltag, Towcester, UK); hamster anti-mouse CD11c mAb (clone N418, AbD Serotec); rabbit anti-annexin V polyclonal Ab (Abcam, Cambridge, UK); rabbit anti-mouse CCL20 polyclonal Ab (R&D Systems, Abingdon, UK); rabbit anti-ubiquitin D polyclonal Ab (Proteintech, Manchester, UK). For the detection of SpiB in paraformaldehyde-fixed sections, antigen retrieval was performed with citrate buffer (pH 7.0, 121 °C, 5 min) prior to immunostaining with sheep anti-mouse SpiB polyclonal antibody (R&D Systems). Unless indicated otherwise, following the addition of primary antibody, species-specific secondary antibodies coupled to Alexa Fluor 488 and Alexa Fluor 555 dyes were used (Invitrogen). Sections were counterstained with either Alexa Fluor 647-conjugated phalloidin or DAPI (ThermoFisher), mounted in fluorescent mounting medium (Dako, Ely, UK) and examined using a Zeiss LSM710 confocal microscope (Zeiss, Welwyn Garden City, UK).

### 2.4 Image analysis

For morphometric analysis, digital microscopy images were analyzed using ImageJ software (http://rsb.info.nih.gov/ij/) as described (Inman et al., 2005). All images were coded and assessed blindly. Background intensity thresholds were first applied using an ImageJ macro which measures pixel intensity across all immunostained and non-stained areas of the images. The obtained pixel intensity threshold value was then applied in all subsequent analyses. Next, the number of pixels of each colour (black, red, green, yellow *etc*.) were automatically counted and presented as a proportion of the total number of pixels in each area under analysis. In each instance, tissues from 4 to 7 mice/group were analyzed. In order to analyse the expression of several parameters in each mouse, multiple sections from 3 to 5 Peyer’s patch were analyzed.

### 2.5 Real-time quantitative PCR (RT-qPCR) analysis of mRNA expression

Total RNA was isolated using RNA-Bee (AMS Biotechnology, Oxfordshire, UK) followed by treatment with DNase I (Ambion, Warrington, UK). First strand cDNA synthesis was performed using 1 μg of total RNA and the First Strand cDNA Synthesis kit (GE Healthcare, Bucks, UK) as described by the manufacturer. PCR was performed using the Platinum-SYBR Green qPCR SuperMix-UDG kit (Invitrogen) and the Stratagene Mx3000P real-time qPCR system (Stratagene, CA, USA). The qPCR primers (Table 1) were designed using Primer-Blast software (de Lau et al., 2012). The cycle threshold values were determined using MxPro software (Stratagene) and normalized relative to *Gapdh*.

### 2.6 Analysis of gene expression in publicly available transcriptomics data sets

The expression levels of the genes encoding the individual NF-κB subunits c-Rel, RelA and RelB (*Rel*, *Rela* and *Relb*, respectively) in the FAE and M cells were compared in publicly available deep CAGE RNA sequence data from the FANTOM consortium (Forrest et al., 2014). The promoter regions of each gene were first identified using ZENBU, the FANTOM5 mouse promoterome viewer (http://fantom.gsc.riken.jp/zenbu). The expression levels of each gene from these promoters were then determined in each of the FAE (datasets CNhs13200 & CNhs13211) and GP2^+^ M cell (datasets CNhs13228, CNhs13231 & CNhs13240) datasets represented using the RLE normalized counts of each CAGE tags mapping to each promoter.

The expression of *Rel*, *Rela*, *Relb* and *Ubd* were also compared in publicly-available gene expression data derived from enriched-FDC performed on Affymetrix mouse genome U74v2 expression arrays (Huber et al., 2005). The expression of *Lta*, *Ltb*, *Tnf* and *Slc7a6* was also compared in publicly-available gene expression data derived from WT and c-Rel-deficient B cells performed on Affymetrix mouse genome 430 2.0 expression arrays (Heise et al., 2014). In each instance the raw data (.cel) files were downloaded and normalized using Robust Multichip Analysis (RMA EXPRESS; http://rmaexpress.bmbolstad.com/), and annotated using the latest library files available from Affymetrix (http://www.affymetrix.com/).

### 2.7 Statistical analyses

All data are derived from 3 to 5 Peyer’s patches from 7 mice/group. Data are presented as mean ± SD. Unless indicated otherwise, differences between groups were analysed by a Student's *t*-test. In instances where there was evidence of non-normality (identified by the Kolmogorov-Smirnov test), data were analysed by a two-tailed Mann-Whitney *U* test. Values of *P* < 0.05 were accepted as significant.

## 3 Results

### 3.1 c-Rel-deficiency does not influence RANKL and RANK expression in Peyer’s patches

**Fig. 1:**
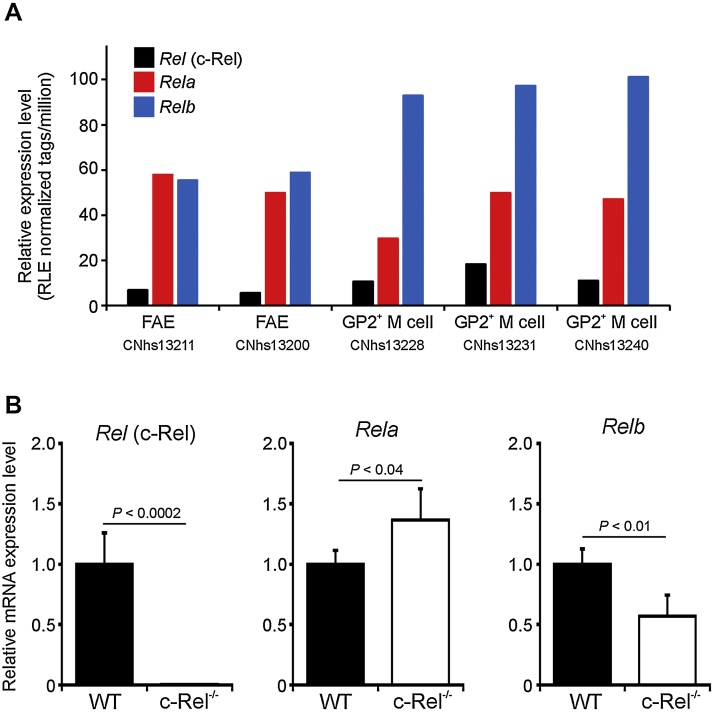
The NF-κB subunits c-Rel, RelA and RelB are expressed in Peyer’s patches in the FAE and M cells. (A) Comparison of *Rel* (which encodes c-Rel), *Rela* and *Relb* mRNA expression in deep CAGE RNA sequence data derived from individual samples of FAE and M cells in the FANTOM Consortium (Forrest et al., 2014) (http://fantom.gsc.riken.jp). (B) RT-qPCR analysis *Rel*, *Rela* and *Relb* expression in Peyer’s patches from c-Rel^−/−^ or wild type (WT) control mice. Gene expression data are normalised so that the mean level in samples from WT mice was 1.0.

First we compared the expression of the genes encoding the individual NF-κB subunits c-Rel, RelA and RelB in the FAE and M cells in publicly available deep CAGE RNA sequence data from the FANTOM consortium (Forrest et al., 2014). This analysis showed that each of these NF-κB subunits were expressed at the mRNA level in the Peyer’s patches in the FAE and by M cells (Fig. 1A). The NF-κB subunits RelA and RelB play an important role in the development of secondary lymphoid tissues, including Peyer’s patches, and deficiency in these genes prevents their development (Yilmaz et al., 2003, Alcamo et al., 2002). Thus it is not possible to study the role of RelA and RelB in the FAE and the M cells within it using RelA- or RelB-deficient mice since they lack Peyer’s patches. The formation of Peyer’s patches in c-Rel^−/−^ mice, in contrast, is not adversely affected (Liou et al., 1999) and were used here to determine the role of c-Rel in M cell differentiation and maturation. As anticipated, RT-qPCR analysis confirmed that *Rel* (which encodes c-Rel) mRNA expression was undetectable in Peyer’s patches from c-Rel^−/−^ mice (Fig. 1B). Although *Rela* and *Relb* mRNA were clearly expressed in Peyer’s patches from c-Rel^−/−^ mice, their levels were significantly different to those expressed in wild type (WT) control mice (Fig. 1B).

**Fig. 2:**
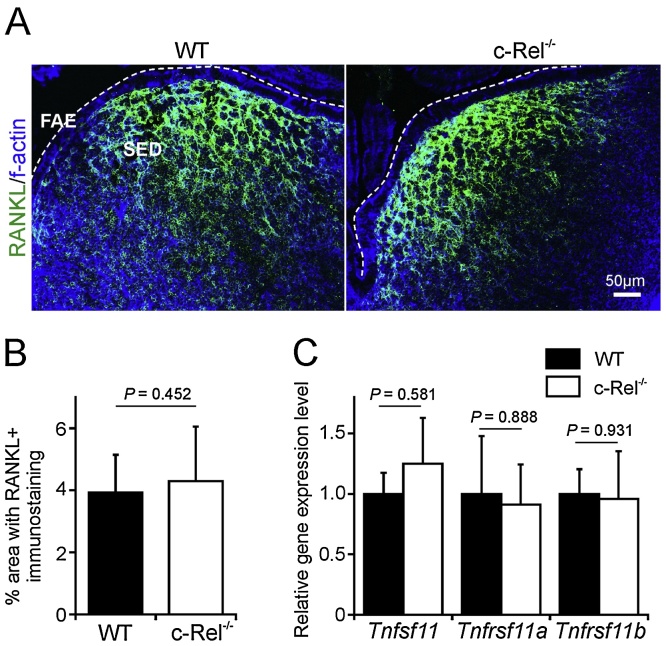
c-Rel-deficiency does not influence RANKL and RANK expression in Peyer’s patches. (A) IHC analysis suggested there was no observable difference in the expression or distribution of RANKL on sub-epithelial dome (SED) stromal cells in Peyer’s patches from c-Rel^−/−^ and wild type (WT) control mice. Broken line indicates the lumenal surface of the follicle-associated epithelium (FAE). Sections are counterstained to detect f-actin (blue). (B) Morphometric analysis confirmed that the magnitude of RANKL-specific immunostaining observed in the SED of Peyer’s patches from WT and c-Rel^−/−^ mice was similar. (C) RT-qPCR analysis suggested there was no significant difference in the expression levels of *Tnfsf11* (RANKL), *Tnfrsf11a* (RANK) or *Tnfrsf11b* (osteoprotegerin) mRNA in Peyer’s patches from c-Rel^−/−^ or WT mice. Gene expression data are normalised so that the mean level in samples from WT mice was 1.0. (For interpretation of the references to colour in this figure legend, the reader is referred to the web version of this article.)

Next we determined whether RANKL expression in Peyer’s patch SED stromal cells was affected in the absence of c-Rel. Immunohistochemical (IHC) analysis showed that high levels of RANKL were expressed on stromal cells in the SED of c-Rel^−/−^ and WT mice (Fig. 2A). Morphometric analysis confirmed that the magnitude of the RANKL immunostaining was similar in Peyer’s patches from each mouse group (Fig. 2B). Similarly, RT-qPCR analysis suggested no significant difference in the expression levels of mRNA encoding RANKL (*Tnfsf11*), RANK (*Tnfrsf11a*) or the RANKL decoy receptor osteoprotegerin (*Tnfrsf11b*) mRNA in tissues from c-Rel^−/−^ mice or WT mice (Fig. 2C). These data show c-Rel-deficiency does not influence the expression of RANKL or RANK in Peyer’s patches.

### 3.2 Effect of c-Rel-deficiency on the differentiation and functional maturation of M cells

**Fig. 3:**
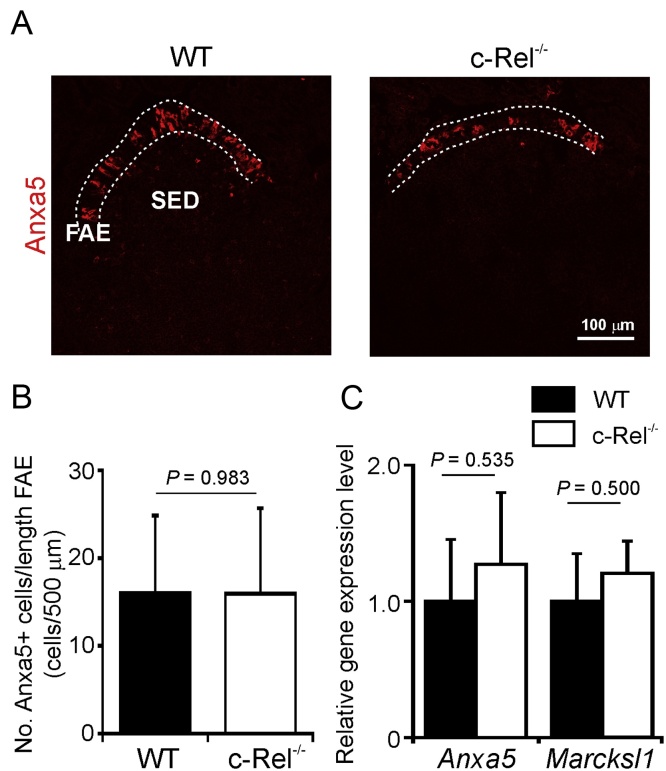
c-Rel-deficiency does not influence expression of the immature M-cell markers Anxa5 and Marcksl1. (A) IHC analysis suggested there was no observable difference in the expression of Anxa5 in the FAE of Peyer’s patches from c-Rel^−/−^ and wild type (WT) control mice. Broken lines indicate the boundaries of the FAE. (B) Morphometric analysis confirmed that the magnitude of Anxa5-specific immunostaining observed in the FAE of Peyer’s patches from c-Rel^−/−^ and WT mice was similar. (C) RT-qPCR analysis suggested there was no significant difference in the expression levels of *Anxa5* or *Marcksl1* mRNA in Peyer’s patches from each mouse group. Gene expression data are normalised so that the mean level in WT was 1.0.

The RANKL-induced process of M-cell differentiation in the FAE can be divided into distinct stages based on the expression of particular sets of proteins (Mabbott et al., 2013, Kanaya et al., 2012, Kimura et al., 2015). Soon after RANKL-stimulation the immature, differentiating, M cells express annexin A5 (Anxa5) and myristoylated alanine-rich C-kinase substrate-like protein 1 (Marcksl1). We therefore first determined whether the differentiation of M cells was adversely affected in the Peyer’s patches of c-Rel^−/−^ mice. IHC analysis showed that the expression of Anxa5 in the FAE of c-Rel^−/−^ mice was similar to that observed in the FAE of WT control mice (Figs. 3A and B). c-Rel-deficiency also did not significantly influence the expression level of *Anxa5* or *Marcksl1* mRNA (Fig. 3C). Together, these data suggest that c-Rel-deficiency does not affect the induction of M-cell differentiation in the FAE of Peyer’s patches.

**Fig. 4:**
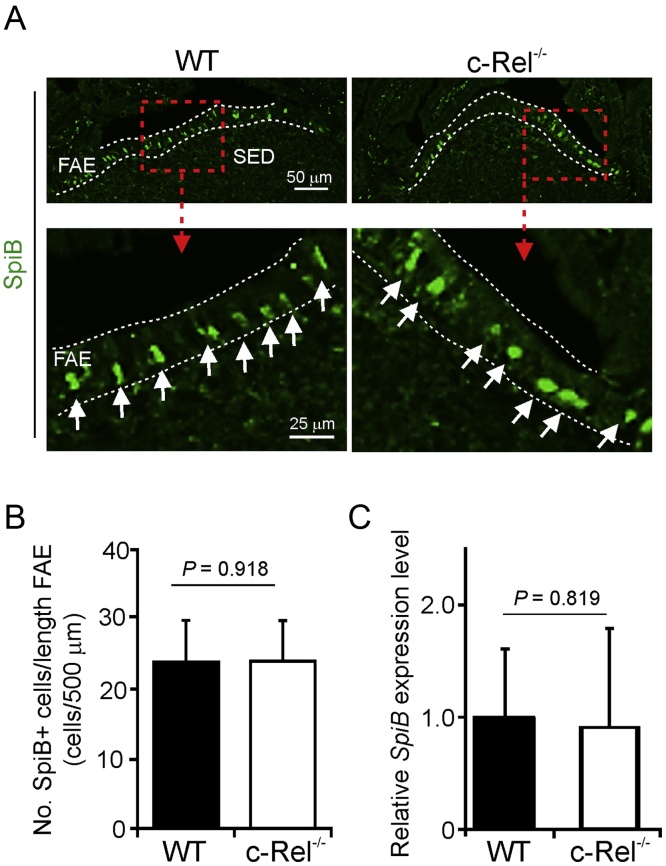
SpiB expression in the FAE is unaffected in the absence of c-Rel. (A) IHC analysis of SpiB expression (green) in the FAE of c-Rel^−/−^ and wild-type (WT) control mice. Boxed areas in upper panels are shown at higher magnification in the lower panels. Broken lines indicate the FAE boundaries. Arrows, Spi-B^+^ cell nuclei in the FAE. (B) Morphometric analysis showed that the number of SpiB^+^ cells in the FAE of c-Rel^−/−^ and WT were similar. (C) RT-qPCR analysis suggested there was no significant difference in the expression of *Spib* mRNA levels in Peyer’s patches from c-Rel^−/−^ or WT mice. Gene expression data are normalised so that the mean level in samples from WT mice was 1.0. (For interpretation of the references to colour in this figure legend, the reader is referred to the web version of this article.)

Expression of the ETS transcription factor SpiB is also rapidly induced in the immature differentiating M cells following RANKL-stimulation (Kanaya et al., 2012, de Lau et al., 2012, Sato et al., 2013), and is essential for their subsequent differentiation into functionally mature M cells (Kanaya et al., 2012, de Lau et al., 2012, Sato et al., 2013). IHC analysis showed that there were similar numbers of SpiB-expressing cells within the FAE of c-Rel^−/−^ and WT mice (Fig. 4A and B). Furthermore, there was no significant difference in the expression levels of *Spib* mRNA in Peyer’s patches from c-Rel^−/−^ or WT mice (Fig. 4C).

**Fig. 5:**
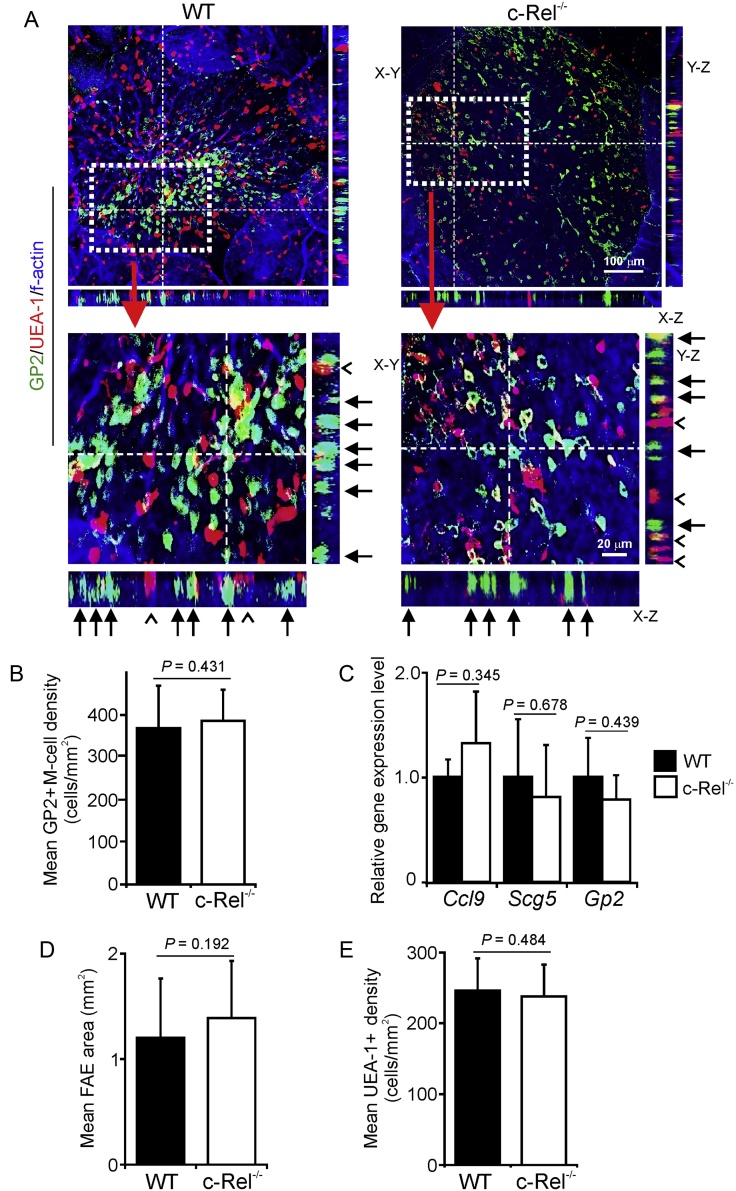
c-Rel-deficiency does not influence the density of mature GP2^+^ M cells in the FAE. (A) Tissues were immunostained to detect GP2 (green), UEA-1 (red) and f-actin (blue). The positions of the X-Z and Y-Z projections of the FAE are indicated by the broken line in the main X-Y images. Closed arrows indicate GP2^+^ M cells with characteristic basolateral pockets. Open arrow-heads indicate GP2^−^UEA-1^+^ goblet cells. The boxed areas in each of the main X-Y images are shown below at higher magnification. (B) Morphometric analysis revealed that the number of GP2^+^ M cells in the FAE of c-Rel^−/−^ and wild type (WT) mice were similar (*P* = 0.431, two-tailed Mann-Whitney *U* test). (C) Comparison of the expression levels of *Gp2*, *Ccl9* and *Sgne1* mRNA in Peyer’s patches of c-Rel^−/−^ and WT mice by RT-qPCR analysis. Gene expression data are normalised so that the mean level in WT mice was 1.0. (D) Morphometric analysis suggested that the size of the FAE in Peyer’s patches of c-Rel^−/−^ and WT mice was similar (*P* = 0.192, two-tailed Mann-Whitney *U* test). (E) c-Rel-deficiency did not influence the density of GP2^−^UEA-1^+^ goblet cells in the FAE (*P* = 0.484, two-tailed Mann-Whitney *U* test). (For interpretation of the references to colour in this figure legend, the reader is referred to the web version of this article.)

Terminally-differentiated mature M cells specifically express glycoprotein 2 (GP2), secretogranin V (Scg5/Sgne1) and the chemokine CCL9, in addition to Anxa5 and Marcksl1 (Hase et al., 2009, Kanaya et al., 2012, de Lau et al., 2012, Wang et al., 2009). Furthermore, the expression of these markers in M cells coincides with the development of their functional ability to transcytose particulate antigens across the FAE (Hase et al., 2009, Kanaya et al., 2012, de Lau et al., 2012). Therefore, we next used whole-mount IHC to compare the density of mature GP2^+^ M cells in the Peyer’s patches of c-Rel^−/−^ and WT mice. Coincident with data above showing that SpiB expression in the FAE was unaffected in the absence of c-Rel, our analysis showed that mature GP2^+^ M cells with characteristic basolateral pockets were abundant in the FAE of Peyer’s patches from c-Rel^−/−^ and WT mice (Fig. 5A, arrows and B). Furthermore, RT-qPCR analysis showed that c-Rel-deficiency did not significantly influence the expression levels mRNA encoding the mature M-cell markers GP2, CCL9 and Scg5 (Fig. 5C). Deficiency in certain NF-κB components can dramatically disturb or inhibit the development of secondary lymphoid tissues, including Peyer’s patches (Yilmaz et al., 2003, Alcamo et al., 2002, Paxian et al., 2002). However, morphometric analysis showed that FAE size was similar in Peyer’s patches of c-Rel^−/−^ and WT mice (Fig. 5D). The density of mucus-secreting goblet cells recognised by their morphology, absence of GP2-expression and binding of the lectin *Ulex europaeus* agglutinin-1 (UEA-1; UEA-1^+^ GP2^−^ cells) was also similar in Peyer’s patches from c-Rel^−/−^ and WT mice (Fig. 5E). Together, these data show that c-Rel-deficiency does not adversely affect the maturation of M cells in Peyer’s patches.

**Fig. 6:**
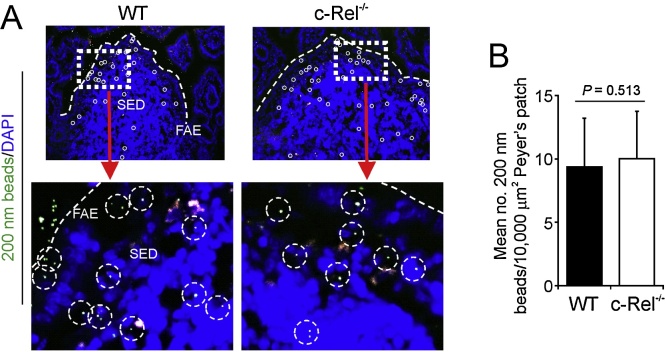
c-Rel deficiency does not influence the uptake of particulate antigen into the Peyer’s patches. (A) c-Rel^−/−^ and wild-type (WT) mice were orally gavaged with 200 nm fluorescent microbeads and 24 h later, the presence of the microbeads in their Peyer’s patches was determined by fluorescence microscopical analysis. FAE, follicle-associated epithelium; SED, sub-epithelial dome; individual beads within the SED are highlighted by broken circles. (B) The number of beads transcytosed across the FAE of c-Rel^−/−^ and WT mice was similar (*P* = 0.513, two-tailed Mann-Whitney *U* test). Data were collected from at least 10 sections from 3 to 5 Peyer’s patches from 4 to 7 mice/group.

We next determined whether the c-Rel deficiency influenced the transcytosis of particulate antigen across the gut lumen. Assessment of the uptake of fluorescent latex beads from the lumen of the small intestine provides a reliable method to quantitatively compare the functional ability of M cells *in vivo* to take up particulate antigen from the gut lumen and transcytose them across the FAE to underlying mononuclear phagocytes in their intraepithelial pockets (Kanaya et al., 2012, Knoop et al., 2009, Donaldson et al., 2015). Here, groups of c-Rel^−/−^ and WT mice were orally gavaged with 1 × 10^12^ 200 nm fluorescent microbeads, and 24 h later the number of microbeads in their Peyer’s patches was quantified by fluorescence microscopy. Our data clearly show that c-Rel deficiency did not significantly impede the uptake of particulate antigen from the gut lumen as abundant fluorescent beads were detected in the SED regions of Peyer’s patches from mice from each group (Fig. 6). Taken together, these data show that c-Rel-deficiency does not adversely affect the functional maturation of M cells in Peyer’s patches.

### 3.3 Effect of c-Rel-deficiency on the expression of FAE-specific genes

**Fig. 7:**
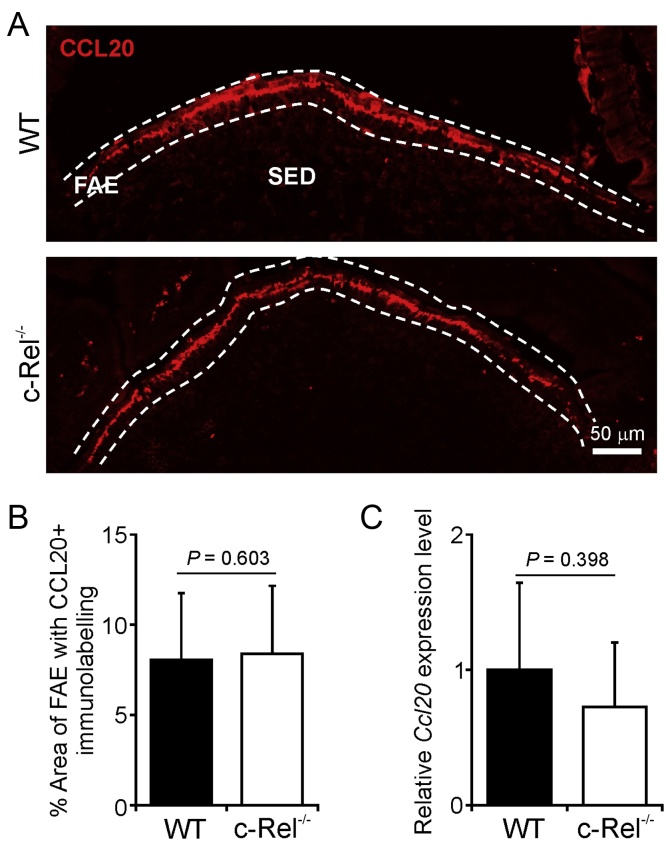
c-Rel-deficiency does not influence CCL20 expression in the FAE. (a) IHC detection of CCL20 (red) in the FAE of c-Rel^−/−^ and wild type (WT) control mice. Broken line indicates the boundaries of the FAE; SED, sub-epithelial dome. (b) Morphometric analysis showed that the magnitude of CCL20-specific immunostaining was similar in the FAE of c-Rel^−/−^ and WT mice. (c) RT-qPCR analysis of *Ccl20* mRNA levels in Peyer’s patches from c-Rel^−/−^ or WT mice. Gene expression data are normalised so that the mean level in WT mice was 1.0. (For interpretation of the references to colour in this figure legend, the reader is referred to the web version of this article.)

Our previous analysis of a cluster of genes which were selectively expressed in the FAE suggested that transcription factor binding sites for the NF-κB transcription factor family members were enriched within the promoter regions of the genes encoding the chemokine CCL20 (*Ccl20*) and ubiquitin D (*Ubd*) (Kobayashi et al., 2012). We therefore determined the effect of c-Rel deficiency on the expression of CCL20 and UBD in the FAE. In WT mice, as anticipated, dense CCL20-specific immunolabeling was detected exclusively in association with the FAE (Fig. 7A) (Kobayashi et al., 2013, Zhao et al., 2003, Ebisawa et al., 2011). Our analysis showed that c-Rel-deficiency did not significantly affect the magnitude and distribution of CCL20 protein or mRNA expression when compared to WT mice (Fig. 7A–C).

**Fig. 8:**
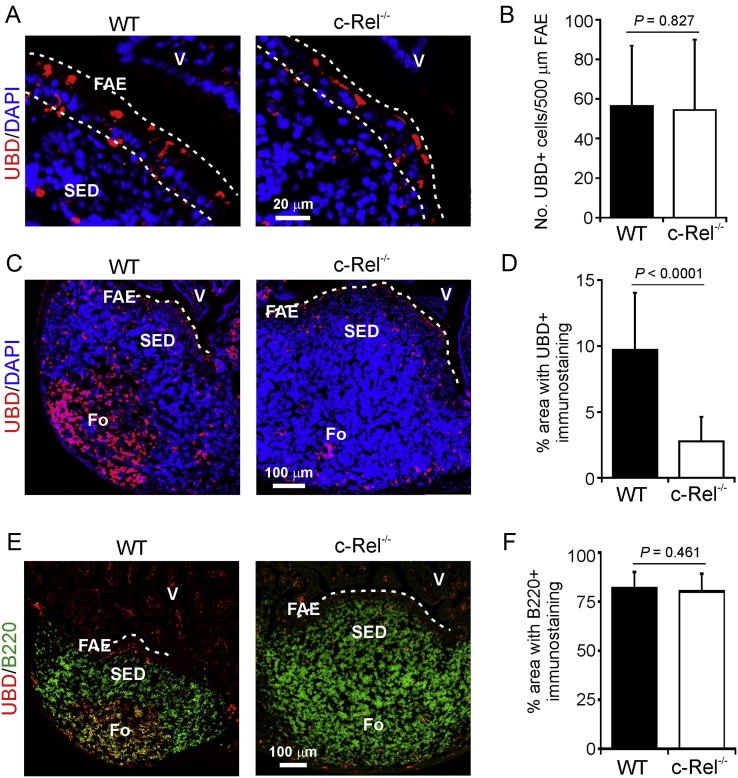
Effect of c-Rel-deficiency on ubiquitin D (UBD) expression in Peyer’s patches. IHC detection of UBD (red) in (A) the follicle-associated epithelium (FAE) and (C) B cell follicles of Peyer’s patches from c-Rel^−/−^ and wild type (WT) control mice. (B and D) Morphometric analysis of the magnitude of UBD-specific immunostaining in (B) the FAE, and (D) B cell follicles of Peyer’s patches from c-Rel^−/−^ and WT mice. (E) IHC analysis of the distribution of B cells (B220^+^ cells, green) and UBD (red) in the Peyer’s patches of c-Rel^−/−^ and WT mice. (F) Morphometric analysis of the magnitude of B220-specific immunostaining in Peyer’s patches from c-Rel^−/−^ and WT mice. Broken lines indicates the boundary of the FAE. Fo, B-cell follicle; SED, subepithelial dome; V, villi. (For interpretation of the references to colour in this figure legend, the reader is referred to the web version of this article.)

In WT mice UBD expression was also detected by IHC in cells throughout the FAE (Fig. 8A) (Kobayashi et al., 2012, Hase et al., 2005). Morphometric analysis suggested that the magnitude of UBD expression in the FAE was likewise similar in Peyer’s patches from c-Rel^−/−^ and WT mice (Fig. 8B). While these data show that c-Rel is dispensable for the expression of CCL20 and UBD in the FAE, a dramatic difference in UBD expression by B cells (B220^+^ cells) within the B cell-follicles was observed. In Peyer’s patches from WT mice UBD was abundantly expressed within the B-cell follicles (Fig. 8C and E). However, in tissues from c-Rel^−/−^ mice the magnitude of UBD in the B-cell follicles was dramatically reduced (Fig. 8C and D). The reduced UBD expression in the Peyer’s patches of c-Rel^−/−^ mice was not due an overall reduction in B cells, since IHC analysis suggested that B cell-densities were equivalent in c-Rel^−/−^ and WT mice (Fig. 8E and F).

### 3.4 Effect of c-Rel deficiency on follicular dendritic cell-status

**Fig. 9:**
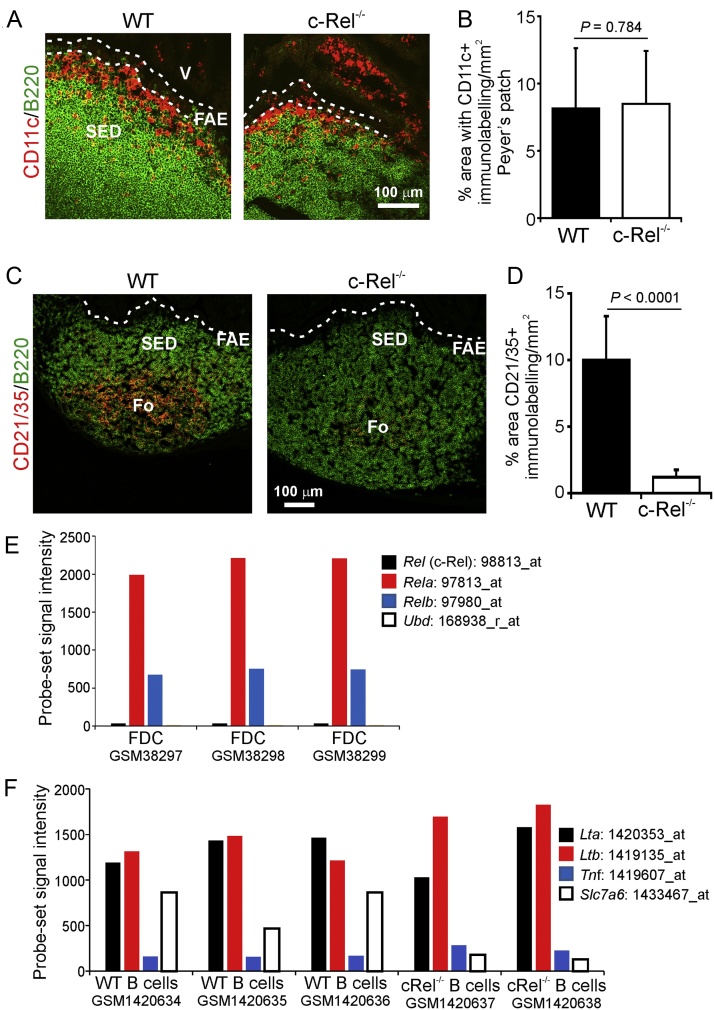
Effect of c-Rel-deficiency on follicular dendritic cell (FDC) status in Peyer’s patches. (A) IHC of the distribution of CD11c^+^ cells (red) in the B cell-follicles (B220^+^ cells, green) of Peyer’s patches from c-Rel^−/−^ and wild type (WT) control mice. (B) Morphometric analysis showed that the magnitude of CD11c-specific immunostaining was similar in tissues from c-Rel^−/−^ and WT mice. (C) IHC analysis of FDC (CD21/35^+^ cells, red) in the B cell follicles (B220^+^ cells, green) of Peyer’s patches from c-Rel^−/−^ and WT mice. (D) Morphometric analysis suggested that the magnitude of C21/35-specific immunostaining was dramatically reduced in tissues from c-Rel^−/−^ mice when compared to WT mice. Broken lines in “A” and “C” indicate the boundary of the follicle-associated epithelium (FAE). Fo, B-cell follicle; SED, subepithelial dome; V, villi. (E) Expression of *Rel*, *Rela*, *Relb* and *Ubd* in publicly-available gene expression data of enriched-FDC performed on Affymetrix mouse genome U74v2 expression arrays (Huber et al., 2005). (F) Comparison of the expression of *Lta*, *Ltb*, *Tnf* and *Slc7a6* in publicly-available gene expression data of c-Rel-deficient and WT B cells performed on Affymetrix mouse genome 430 2.0 expression arrays (Heise et al., 2014). The individual Affymetrix probe sets for each gene analysed are indicated. GSMXXXX, accession codes of each data set used. (For interpretation of the references to colour in this figure legend, the reader is referred to the web version of this article.)

We next determined the effect of c-Rel-deficiency on other cell populations in Peyer’s patches. Our IHC analysis suggested that the density and distribution of conventional dendritic cells (DC; CD11c^+^ cells) was similar in tissues from WT and c-Rel^−/−^ mice (Fig. 9A and B). These data are consistent with data from a previous study which showed that conventional DC development and function was normal on c-Rel^−/−^ mice (Quaaz et al., 2002). However, a dramatic reduction in the status of CD21/35-expressing stromal-derived follicular dendritic cells (FDC; (Krautler et al., 2012)) was observed in the Peyer’s patches of c-Rel^−/−^ mice (Fig. 9C and D). Our retrospective analysis of publicly-available gene expression data of mRNA prepared from enriched FDC (Huber et al., 2005), revealed that whereas *Rela* and *Relb* were highly expressed, *Rel* was only expressed at background levels, if at all, in enriched FDC (Fig. 9E). These data are consistent with data from an earlier study which suggested that c-Rel was expressed by lymphocytes, but not by FDC (Feuillard et al., 1994). Furthermore, *Ubd* expression was also undetectable in enriched FDC (Fig. 9E). This implied that the effects of c-Rel-deficiency on FDC were indirect, and most-likely due to the effects of c-Rel-deficiency on germinal centre B cells. Constitutive stimulation from lymphocyte-derived lymphotoxin (LTα_1_β_2_) and TNFα are essential to maintain FDC in their differentiated state (Mackay and Browning, 1998). However, our retrospective comparison of *Lta*, *Ltb* and *Tnf* expression in mRNA derived from c-Rel-deficient B cells (Heise et al., 2014) showed they were each expressed at similar levels to WT B cells (Fig. 9F). Together, these data suggest that the effects of c-Rel-deficiency on FDC status were not due to a specific absence of c-Rel or UBD expression in FDC (Fig. 9E) or the reduced expression of B cell-derived LTα_1_β_2_ or TNFα (Fig. 9F). The expression of genes encoding metabolic regulators such as *Slc7a6* is reduced in c-Rel-deficient B cells (Fig. 9F; (Heise et al., 2014)). Thus it is plausible that the reduced FDC status in c-Rel^−/−^ mice is an indirect consequence of the significantly reduced metabolic activities of their germinal centre B cells (Heise et al., 2014).

## 4 Discussion

Here we show that expression of the NF-κB family transcription factor member c-Rel is dispensable for the differentiation and functional maturation of M cells. Stimulation from SED stromal cells *via* the cytokine RANKL is essential for the induction of M-cell differentiation in the FAE (Knoop et al., 2009). Although RANKL-stimulation can induce the nuclear translocation of c-Rel (Ruocco et al., 2005, Kang et al., 2003), c-Rel-deficiency did not influence the expression of RANKL or RANK in Peyer’s patches, or the RANKL-mediated induction of M-cell differentiation in the FAE. Expression of SpiB, the master regulator of M-cell maturation (Kanaya et al., 2012, de Lau et al., 2012, Sato et al., 2013), was also unaffected in the FAE in the absence of c-Rel. As a consequence, the densities of functionally mature M cells in the Peyer’s patches of c-Rel^−/−^ and WT mice were similar. Mature M cells express GP2 highly on their apical surfaces. This protein has been shown to selectively bind FimH^+^ bacteria such as *Eschericia coli* and *Salmonella* Typhimurium, and enable their specific uptake and transcytosis by M cells (Hase et al., 2009). In the current study the uptake of 200 nm beads and the expression of GP2 mRNA and protein were not affected in the absence of c-Rel, suggesting that the transcytosis of particulate antigens and bacteria is also unlikely to be adversely affected by c-Rel-deficiency.

Signalling *via* NF-κB can occur *via* the canonical or non-canonical activation pathways (Oeckinghaus et al., 2011). The canonical NF-κB pathway concludes with the activation of dimers of the RelA, c-Rel and p50 NF-κB subunits, whereas the non-canonical NF-κB activation pathway, in contrast, activates dimers of the p100 and RelB NF-κB subunits. The treatment of mice with recombinant RANKL induces the nuclear translocation of RelB in the gut epithelium (Kimura et al., 2015), and has been proposed to play a role in the initiation of M-cell differentiation. Although our data do not exclude a role for the RelA and p50 NF-κB subunits, our data together with those from independent studies of M cell-differentiation using either *in vitro* enteroids (Wood et al., 2016) or exogenous RANKL-treatment *in vivo* (Kimura et al., 2015), suggest a requirement for the non-canonical NF-κB activation pathway in RANKL-mediated M-cell differentiation.

In the intestine, high-affinity antigen-specific IgA antibodies are produced by B cells through a process termed affinity maturation which occurs within the germinal centres of the B-cell follicles in the GALT (Shikina et al., 2004, Barone et al., 2011, Knoop and Newberry, 2012). Antigen-stimulated B cells enter the germinal centre, where they proliferate and undergo hypermutation within their immunoglobulin genes. The B cells which weakly bind antigen undergo negative selection in the germinal centre and are eliminated by apoptosis. Conversely, those B cells which contain mutations which improve the antigen affinity of the expressed antibody receive rescue signals. These positively selected B cells undergo further rounds of hypermutation and selection before ultimately developing into antibody secreting plasma cells and exiting the germinal centre. This selection is mediated by interactions between T follicular helper cells and the germinal centre B cells with the greatest surface expression of antigen-derived peptide bound to major histocompatibility complex (MHC) class II (Shulman et al., 2014). The expression level and turnover of MHC class II in germinal centre B cells is dynamically regulated by changes in ubiquitination (Bannard et al., 2016). The addition of ubiquitin chains to MHC class II by the E3 ligase March1 targets the protein for lysosomal destruction and prevents it from recycling back to the cell surface (Cho et al., 2015). These changes in MHC class II turnover on germinal centre B cells play an important role in promoting productive interactions with T follicular helper cells during the selection process (Bannard et al., 2016). In the current study we show that UBD expression was dramatically reduced in the B-cell follicles of Peyer’s patches from c-Rel^−/−^ mice. This observation is consistent with the demonstration that the maintenance and differentiation of the germinal centres within the B-cell follicles is dependent on c-Rel expression (Heise et al., 2014). Indeed, B cells from c-Rel-deficient mice display reduced expression of genes encoding regulators of cellular metabolism that are necessary to meet the high energy requirements of the rapidly proliferating germinal centre B cells (Heise et al., 2014).

FDC are a unique population of stromal cells which reside within the B cell-follicles and germinal centres of secondary lymphoid tissues (Krautler et al., 2012). These cells express complement receptors (CR; CR1/CD35 and CD2/CD21) and immunoglobulin-receptors which enable them to trap and retain antigen-containing immune complexes on their surfaces (Fang et al., 1998). Antigen-retention by FDC is important for the promotion of immunoglobulin-isotype subclass switching, positive selection of high affinity B cells and long-term maintenance of immunological memory (Victoratos et al., 2006, Heesters et al., 2014). Further analysis of the B cell-follicle regions of Peyer’s patches from c-Rel^−/−^ mice revealed a dramatic reduction in FDC status. However, FDC unlike lymphocytes are not considered to express c-Rel (Feuillard et al., 1994), and our retrospective analysis of mRNA from enriched-FDC (Huber et al., 2005), demonstrated that *Rel* and *Ubd* expression were undetectable in FDC. These data imply that the effects of c-Rel-deficiency on FDC were indirect, and most-likely due to the role of c-Rel-deficiency in the maintenance of germinal centre B cells (Heise et al., 2014). Analysis of publicly-available gene expression data from c-Rel-deficient B cells (Heise et al., 2014) indicated that the expression of the essential cytokines required for the constitutive maintenance of FDC in their differentiated state (LTα_1_β_2_ and TNFα; (Mackay and Browning, 1998)) were unlikely to be adversely affected by c-Rel-deficiency. The precise effect of c-Rel-deficiency on FDC is uncertain since an absence of germinal centres themselves in interleukin-6-deficient mice does not cause a similar reduction in FDC status (Mabbott et al., 2000). The reduced germinal centre formation in c-Rel^−/−^ mice is associated with a failure of their B cells to activate a metabolic program that provides sufficient energy to promote their growth and proliferation (Heise et al., 2014). We therefore speculate that the significantly reduced metabolic activities of c-Rel-deficient germinal centre B cells may render them unable to provide sufficient support to maintain FDC in their differentiated state.

Data from several independent studies show that the specific targeting of vaccine antigens to M cells is an effective means of inducing an antigen-specific mucosal immune response (Nochi et al., 2007, Shima et al., 2014, Singh et al., 2015). Furthermore, the increased abundance of M cells in the gut epithelium after systemic RANKL treatment has been reported to enhance the uptake of orally-administered antigens from the gut lumen and in doing so improve antigen-specific mucosal immunity (Maharjan et al., 2016). Thus a thorough understanding of the molecular mechanisms which underpin the RANKL-mediated induction of M-cell differentiation in the intestine may lead to the identification of novel mechanisms to enhance mucosal immunity or the development of strategies to block transmission of important orally-acquired infections.

## Conflict of interest

None.

## Figures and Tables

**Table 1:** Primers used for RT-qPCR analysis.

Gene	Forward primer (5′ to 3′)	Reverse primer
*Anxa5*	TTTCCGTTGCACGGAGTTGT	TTTCCTGGCGCTGAGCATT
*Ccl9*	TACTGCCCTCTCCTTCCTCA	TTGAAAGCCCATGTGAAACA
*Ccl20*	CGACTGTTGCCTCTCGTACA	AGCCCTTTTCACCCAGTTCT
*Gapdh*	GATACTGCACAGACCCCTCCA	GCAGTTCCGGTCATTGAGGTA
*Gp2*	CTGCGTTCTGACACTGGTATCTC	GATTCTGGCAGGGATCAAAGC
*Marcksl1*	TTTTGCCCTCCTGTGGATTCT	CCACTAGGCACAGCACAAGAGA
*Rel*	CCTTCCAAATGCTGGGGCTA	CCAGACAGGGTGGTGTTACC
*Rela*	AACTCACCAGCAGTTCGAGG	CGAGATCTGACGCCCTCTTC
*Relb*	TACTTGTCTCCCTAGCCCCC	GACAGCCTGGTGTACGTTGA
*Scg5*	ACGGTTAAAAATGGCCTCAAGG	AAGGACCCAGATGCTGAAGACC
*SpiB*	AGCGCATGACGTATCAGAAGC	GGAATCCTATACACGGCACAGG
*Tnfsf11*	GAAGGCTCATGGTTGGATGTGG	GTGACTTTATGGGAACCCGATG
*Tnfrsf11a*	CTGCCTCTGGGAACGTGACTGG	GGCTGACATACACCACGATG
*Tnfrsf11b*	CACCTTGAAGGGCCTGATGT	TTTTGGGAAAGTGGGATGTTTT
*Ubd*	GATTGACAAGGAAACCACTATCCA	ACAAGGGCAGCTCTTCATCAC
